# Cascading Alignment for Unsupervised Domain-Adaptive DETR with Improved DeNoising Anchor Boxes

**DOI:** 10.3390/s22249629

**Published:** 2022-12-08

**Authors:** Huantong Geng, Jun Jiang, Junye Shen, Mengmeng Hou

**Affiliations:** 1 School of Computer Science, Nanjing University of Information Science and Technology, Nanjing 210044, China; 2 School of Information Technology, Jiangsu Open University, Nanjing 210036, China

**Keywords:** object detection, detection transformer, domain adaptation, DINO

## Abstract

Transformer-based object detection has recently attracted increasing interest and shown promising results. As one of the DETR-like models, DETR with improved denoising anchor boxes (DINO) produced superior performance on COCO val2017 and achieved a new state of the art. However, it often encounters challenges when applied to new scenarios where no annotated data is available, and the imaging conditions differ significantly. To alleviate this problem of domain shift, in this paper, unsupervised domain adaptive DINO via cascading alignment (CA-DINO) was proposed, which consists of attention-enhanced double discriminators (AEDD) and weak-restraints on category-level token (WROT). Specifically, AEDD is used to aggregate and align the local–global context from the feature representations of both domains while reducing the domain discrepancy before entering the transformer encoder and decoder. WROT extends Deep CORAL loss to adapt class tokens after embedding, minimizing the difference in second-order statistics between the source and target domain. Our approach is trained end to end, and experiments on two challenging benchmarks demonstrate the effectiveness of our method, which yields 41% relative improvement compared to baseline on the benchmark dataset Foggy Cityscapes, in particular.

## 1. Introduction

As the fundamental task of computer vision (CV), object detection, which involves two sub-tasks: classification versus regression, is widely used in automatic driving [1], face recognition [2], crowd-flow counting [3], and target tracking [3], etc. Over the past decade, classical convolution-based object-detection algorithms have made significant progress. Derived methods consist of one-stage methods, such as the YOLO series [4,5,6,7], and two-stage methods, such as the RCNN series [8,9,10,11,12]. Recently, transformer-based models have attracted increasing attention in CV. As a new paradigm for object detection, detection transformer (DETR) [13] eliminates the need for hand-designed components and shows promising performance compared with most classical detectors based on convolutional architectures due to the processing of global information performed by the self-attention [14]. In the ensuing years, many improved DETR-like methods [15,16,17] have been proposed to address the problems that slow the training convergence of DETR and the meaning of queries. Among them, DETR with improved denoising anchor boxes (DINO) [18] became a new state-of-the-art approach on COCO 2017 [19], proving that transformer-based object-detection models can also achieve superior performance.

Deep neural networks training is extremely dependent on external manual annotation data whose training set and validation set are supposed to be independent and identically distributed. Data labeling is time-consuming and the process can be costly; while some public benchmarks [19,20] already exist, they only cover a limited number of scenarios. In general, the labeled training data is known as the source domain, and the unlabeled validation data, which has a large distribution gap from the training data, is termed the target domain. When applied to the target domain with varying object appearance, altering backgrounds and changing illumination, etc., the performance of the model trained on the source domain would suffer dramatic degradation. To solve the domain shift problem between two domains and to avoid expensive laborious annotations, numerous domain-adaptive methods have been proposed for object detection. Most existing works [21,22,23,24] have achieved significant progress in improving cross-domain performance; universally, these specific methods are based on Faster RCNN [24],YOLOv5 [25] and FCOS [26,27]. Although considerable progress has been made, they complicate network design, and cannot fully utilize synergistic relationships between different network components. Compared with the well-established CNN-based detectors, how to develop efficient domain adaptation methods to enhance the cross-domain performance of DETR-like detectors remains rarely explored. The design draws on DN-DETR [17], DAB-DETR [16], and deformable DETR [15], with DINO achieving an exceptional result on public datasets. However, as with previous object detectors, it cannot be directly applied to new scenarios when variations in environmental conditions change, which results in significant performance degradation.

**Figure 1:**
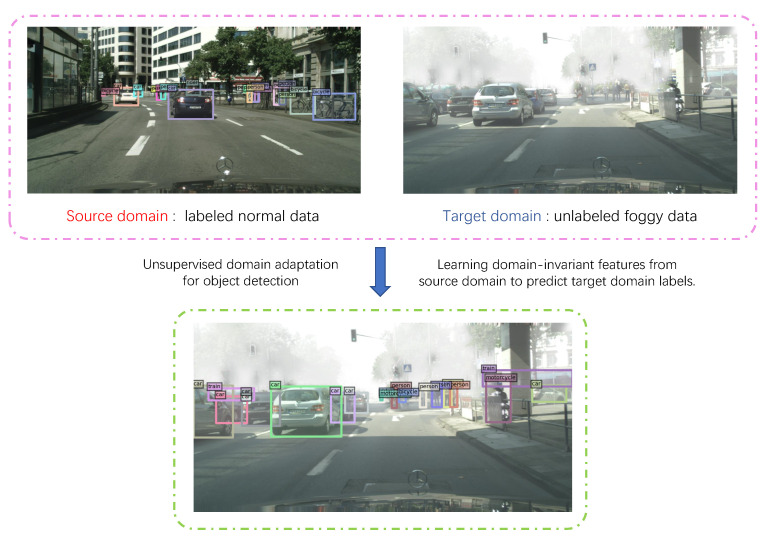
Unsupervised domain-adaptation approach for object detection in foggy scenes. Given a source domain (normal data) with bbox labels and a target domain (foggy scenes) with no annotation. Our goal is to train a model to predict bbox labels of the target domain.

**Figure 2:**
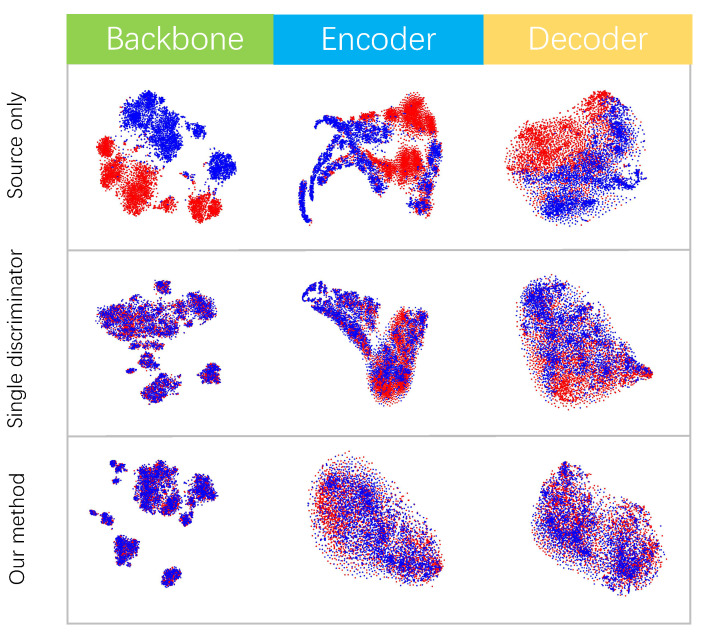
T-SNE [29] visualization of features extracted by DINO [18], single discriminator version and our method. Both methods are built on ResNet-50 [30] backbone and evaluated on the Cityscapes [31] to Foggy Cityscapes [32] scenario (red: Cityscapes; blue: Foggy Cityscapes). Since they contain spatial information, the features from the encoder and decoder do not have a typical cluster attribute.

This work aims to train DINO on the labeled source domain so that it can be applied to the unlabeled target domain, as shown in Figure 1. As a pioneering work in domain adaptation for object detection, DAF [24] introduced adversarial training by adding domain discriminators to allow the model to learn domain-invariant features. In initial attempts, this paper emulates previous work, an existing domain-adaptation method [28] based on the adversarial paradigm with a single discriminator was directly involved. While achieving a considerable performance gain, there is still a significant deviation from the result by training on labeled data in the target domain. Figure 2 shows the distribution of features extracted by DINO, the single discriminator version and our method. For DINO trained on a source domain only, the features extracted by the backbone, encoder and decoder can all be easily separated by domain. This means the models trained on the source domain do not transfer well to the target domain. For the single-discriminator version, while the source and target features extracted by the backbone are aligned, the features from the transformer, encoder and decoder are not aligned properly, which substantially affects the model’s performance. This visualization suggests that it is challenging to learn the domain-invariant features when migrating a single discriminator for domain-adaptive classification tasks into object-detection models such as DINO directly. We began to re-examine the adversarial learning process. Since this weak discriminator is readily tricked, its loss drops dramatically in the middle of training. Furthermore, the model may acquire few domain-invariant features.

To tackle the above problem, a novel cascading alignment strategy was proposed for learning domain-invariant features and applying them to the DINO; then, cascading alignment DINO (CA-DINO), a simple yet effective DETR-like detector, was further designed. CA-DINO consists of two key components: attention-enhanced double discriminators (AEDD) and weak-restraints on category-level token (WROT). Concretely, AEDD contains two parameter-independent discriminators with attention enhanced, which act on the second-last and third-last layer of the backbone, respectively, to learn the domain-invariant features via adversarial training. The backbone containing domain-invariant features is of great help to the unsupervised training of the encoder and decoder, because usually the decoder is more biased towards the source domain with supervised training. A well-aligned backbone could guide the transformer encoder and decoder during training. Compared to the original discriminator, the capacity of discrimination between two domains is considerably improved by AEDD, which makes it less conceivable it will be easily deceived. The introduction of two discriminators for adversarial training leads to instability in training. It makes it difficult for the model to converge in the right direction, making both fine tuning and end-to-end training challenging. Motivated by these findings, a weak constraint based on the statistical method was proposed to regularize the category-level token produced by the transformer encoder and decoder and increase their discriminability for robust object detection.

Overall, the collaboration of these two components results in the proper alignment of domain-invariant features. Compared to other models, our method produced superior outcomes and experiments on two challenging benchmarks, demonstrating that our strategy considerably improves the cross-domain performance of DINO and outperforms various competitive approaches.

The main contributions of this paper are as follows:

- We observe that a weak discriminator is a primary reason why alignment of feature distribution on the backbone yields only modest gains and propose AEDD. It directly scopes the backbone to alleviate the domain gaps and guide the ascension of the cross-domain performance of the transformer encoder and decoder.
- A novel weak-restraints loss is proposed to regularize further the category-level token produced by the transformer decoder and boost its discriminability for robust object detection.
- Extensive experiments on challenging domain adaptation scenarios verify the effectiveness of our method with end-to-end training.

## 2. Related Work

### 2.1. Object Detection

Object detection is a crucial challenge in CV. Representative object detectors based on deep learning may be broadly classified as either two-stage or one-stage approaches. Specifically, in two-stage detectors such as Faster RCNN [10], a region proposal network is designed to propose candidate object bounding boxes, and a region of interest (ROI) pooling operation retrieves the features from each candidate box for the following classification and regression tasks. Typically, they are accompanied by outstanding performance. One-stage detectors, such as YOLO [4], suggest predicted boxes straight from the input without an ROI pooling phase, making them time-efficient and suitable for real-time devices.

Typically, the performance of these models is significantly influenced by hand-designed components, such as anchor generation, for which prior knowledge about the task needs to be explicitly encoded alongside non-maximum suppression [33]. To simplify these processes, DETR [13] views object detection as a direct-set prediction issue and designs an end-to-end architecture based on the transfomer [14]. The following variants [34,35,36], Deformable DETR [15], performs a (multi-scale) deformable attention module, an efficient attention mechanism, which achieves superior performance to DETR and considerably increases the convergence speed of the model. DAB-DETR [16] demonstrates that the primary reason for the sluggish convergence of DETR is that its decoder is challenging to train and proposes a method of using anchors as a query to provide better prior spatial knowledge for the model and speed up the convergence of decoder. DN-DETR [17] indicates that the instability of bipartite graph matching may cause slow convergence and proposes integrating denoising training to accelerate convergence and improve performance. Based on prior research, improving the denoising training, query initialization, and box prediction of DINO [18] considerably enhances both the training efficiency and the final detection performance.

### 2.2. Pipeline of DINO

Like other DETR-like models, DINO generally consists of three parts: the backbone for extracting low-level features, the transformer encoder and decoder for modeling sequence features, and multiple prediction heads for making predictions.

Given an image, the backbone extracts the representation of multi-scale features ${\left\{f_{map}^{l}\right\}}_{l=1}^{L}$, where $f_{map}^{l}\in R^{B\times H^{l}\times W^{l}\times C^{l}}$ denotes the $l^{th}$ feature map, and B denotes batch size. Then these are fed with hierarchical features into the deformable transformer encoder with corresponding positional embeddings to attain refined image sequence features ${f_{s}}^{enc}$, where ${f_{s}}^{enc}\in R^{B\times N\times C}$, N = $\sum_{l=1}^{L}H^{l}W^{l}$, and C refers to the number of channels. Subsequently, a mixed query selection approach is used to initiate anchors as positional queries and add learnable content queries to deformable transformer decoder along with the sequence features of the encoder outputs. Finally, the feedforward neural network predicts classification probability vectors and bounding boxes based on the output of each deformable transformer decoder layer with denoising training approach.

DINO uses the L1 loss [10] and GIOU [37] loss for regression and focal loss [38] for classification and adds additional interim losses after the query selection. $\ell_{det}$ denotes the supervised loss on the source domain.

### 2.3. Domain Adaptation for Object Detection

Domain-adaptive object detection, which seeks to train the detector on the source domain and then apply it to the target domain, has attracted growing interest in recent years. As the pioneering work in adapting domain-adaptive techniques to object detection, DA Faster R-CNN [24] proposes a joint adaptation, which consists of an image-level adaptation module and an instance-level adaptation module to alleviate the performance deterioration caused by domain shift. Inspired by this, SWDA [23] proposes a weak alignment model to align the similar overall feature, and an alignment model to enhance the local sensing field of the feature map based on the discovery of different background layouts of other domains. D-adapt [39] proposes decoupled adaptation, which decouples adversarial adaptation from detector training and introduces a bounding-box adaptor to improve localization performance.

With the extensive use of a transformer in object detection, the DETR-like domain-adaptive object detector has also produced some remarkable outcomes. SFA [40] proposes a novel sequence-feature-alignment method designed for DETR-like models to extract the domain-invariant features of sequence features, as well as a binary matching consistency loss to enhance the robustness of the model further.

In this paper, CA-DINO adopts adversarial learning as the primary mechanism and aims to improve the cross-domain performance of DINO, which is still unexplored.

## 3. Methods

### 3.1. Framework Overview

**Figure 3:**
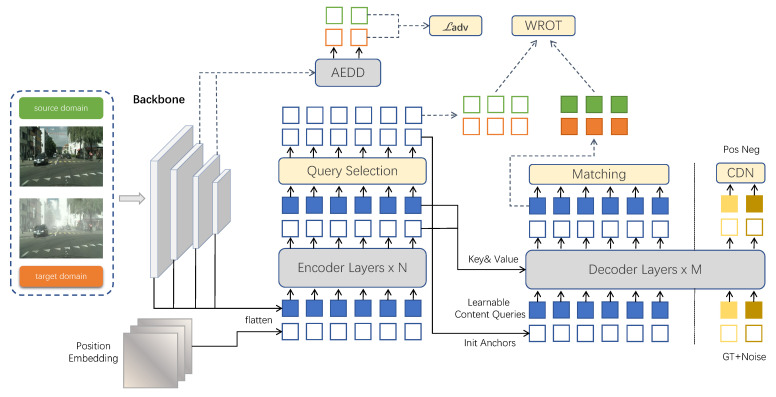
Diagram of CA-DINO for domain-adaptive detection. AEDD aligns the output features of backbone to tackle global and local domain gaps. Moreover, WROT is proposed to improve the performance of DINO on the target domain.

Figure 3 depicts the overall architecture of CA-DINO which introduces AEDD for optimal-feature alignment and WROT for minimizing the difference in second-order statistics between the source and target category-level token. The training data contains both labeled source data $D_{s}={\left\{\left(x_{s}^{i},y_{s}^{i}\right)\right\}}_{i=1}^{N_{s}}$ and unlabeled target data $D_{t}={\left\{x_{t}^{i}\right\}}_{i=1}^{N_{t}}$, where, $N_{s}\left(N_{t}\right)$ represents the number of samples in dataset $D_{s}\left(D_{t}\right)$, $y_{s}^{i}$ represents the labels of the sample image $x_{s}^{i}$, and $D_{t}$ does not contain label $y_{t}^{i}$ which corresponds to sample image $x_{t}^{i}$. Given a pair of images $x_{s}\in D_{s}$ and $x_{t}\in D_{t}$, backbone produced feature maps ${\left\{f_{map_{s}}^{l}\right\}}_{l=1}^{L}$ and ${\left\{f_{map_{t}}^{l}\right\}}_{l=1}^{L}$, then fed to the encoder to obtain latent features $f_{s}^{enc}$ and $f_{t}^{enc}$. After mixed query selection, the selected features $f_{enc}^{obj}$ were used for WROT. These selected features were fed to an auxiliary detection head to obtain predicted boxes, which were used to initialize reference boxes. Additionally, $\left(f_{map_{s}}^{L-1},f_{map_{s}}^{L-2}\right)$ and $\left(f_{map_{t}}^{L-1},f_{map_{t}}^{L-2}\right)$ will be supplied into the AEDD to calculate loss $\ell_{adv}$ for adversarial feature alignment. With the initialized anchors and the learnable content queries, the sequence features $f_{s}^{enc}$ and $f_{t}^{enc}$ are also fed to the deformable transformer decoder to predict a set of bounding boxes and pre-defined semantic categories $f_{dec}^{obj}$, which will be used to calculate a detection loss $\ell_{det}$. $\ell_{coral}$ is constructed from $f_{enc}^{obj}$ and $f_{dec}^{obj}$ to minimize the difference between source and target correlation.

### 3.2. Attention-Enhanced Double Discriminators

**Figure 4:**
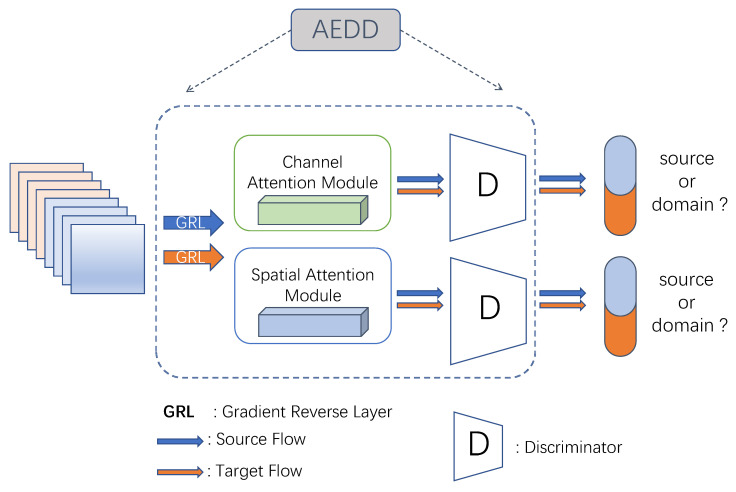
The architecture of AEDD. The discriminator *D* is trained end to end for discrimination from two domains.

Domain-invariant features from the backbone are essential for detection transformers to alleviate the domain shift problem. As in Deformable DETR, DINO applies the multi-scale backbone features to enhance the detection performance for small objects. The structure of AEDD is shown in Figure 4. Gradient reversal layer (GRL) [28] is adopted to enable the gradient of $L_{adv}$ to be reversed before back-propagating to backbone.

To distinguish the feature distribution between source and target domains in different perspectives, the backbone was made to learn domain-invariant representations to fool the discriminator; the features of different domain $\left(f_{map}^{L-1},f_{map}^{L-2}\right)$ were fed into AEDD, which contains two parameter-independent domain discriminators with spatial and channel attention-enhancement:

(1) $$P=F_{sig}\left(D_{1}\left(f_{map}^{L-1}\right),D_{2}\left(f_{map}^{L-2}\right)\right)$$

where $F_{sig}\left(\right)$ is an activation function to limit *P* in [0, 1], $D_{1}$ and $D_{2}$ denote those two discriminators with convolutional block attention module (CBAM) [41] included. The structure of these two discriminators can be implemented in different ways that slightly impact the final result. In this paper, their implementation is generally based on DANN [42]. After adding CBAM, the discriminator, which acts on the antepenultimate layer of the backbone, does not flatten the domain feature into a two-dimensional vector while directly regularising feature maps for better domain discrimination.

The standard adversarial formulation $L_{adv}$ can be formulated as follows:

(2) $$\ell_{adv}=-\left[d\log P_{s}+\left(1-d\right)\log\left(1-P_{s}\right)+\left(1-d\right)\log P_{t}+d\log\left(1-P_{t}\right)\right]$$

where *d* is the domain label, which values 0 for source domain and 1 for target domain. Both source source and target source $\left(P_{s},P_{t}\right)\in P$ are utilized to compute adversarial loss.

### 3.3. Weak Restraints on Category-Level Token

Deep CORAL [43] is a simple yet effective unsupervised domain-adaptation method which aligns correlations of layer activations in the deep neural network for classification. Inspired by this, WROT extends it to the category-level token to close domain gaps at the instance level. Specifically, each category token $f_{enc}^{obj}\in R^{B\times N_{q}\times N_{c}}$ and $f_{dec}^{obj}\in R^{B\times N_{q}\times N_{c}}$ are flattened to form a one-dimensional sequence $z\in R^{N\times N_{c}}$, where $N_{q}$ means the number of queries, $N_{c}$ indicates the number of categories, and *N* denotes $B\cdot N_{q}$; then, the covariance matrices of the source and target data $C_{S}$ and $C_{T}$ are given by:

(3) $$C_{S}=\frac{1}{N-1}\left(z_{S}^{⊤}z_{S}-\frac{1}{N}{\left(1^{⊤}z_{S}\right)}^{⊤}\left(1^{⊤}z_{S}\right)\right)$$

(4) $$C_{T}=\frac{1}{N-1}\left(z_{T}^{⊤}z_{T}-\frac{1}{N}{\left(1^{⊤}z_{T}\right)}^{⊤}\left(1^{⊤}z_{T}\right)\right)$$

where 1 is a column vector in which each element is 1. The $\ell_{coral}$ is defined for measuring distance between the second-order statistics (covariances) of the source and target features:

(5) $$\ell_{coral}=\frac{1}{4d^{2}}{∥C_{S}-C_{T}∥}_{F}^{2}$$

where $∥\dot{}{∥}_{F}^{2}$ denotes the squared matrix frobenius norm and d denotes feature dimension. WROT constrains the category-level token of transformer encoder, and the performance of DINO in the target domain is improved by it.

### 3.4. Total Loss

To summarize, the final training objective of CA-DINO is defined as:

(6) $$\ell =\ell_{det}+\lambda_{adv}\ell_{adv}+\lambda_{coral}\ell_{coral}$$

where $\lambda_{adv}$ and $\lambda_{coral}$ are weights that trade off the adaptation. These three losses constitute counterparts and reach an equilibrium at the end of training, where it is anticipated that the features would perform well on the target domain.

## 4. Experiments

In this section, comprehensive experiments on many cross-domain object-detection scenarios demonstrate the effectiveness of CA-DINO. Ablation studies and visualization analysis validate that our design makes DINO capable of detection in the target domain.

### 4.1. Datasets

In these experiments, the following three public datasets will be employed: Cityscapes [31], Foggy Cityscapes [32] and Sim10k [44], which are detailed as follows.

- Cityscapes [31] has a subset called leftImg8bit, which contains 2975 images for training and 500 images for evaluation with high-quality pixel-level annotations from 50 different cities; consistent with previous work [40], the tightest rectangles of object mask will be used to obtain bounding-box annotation of 8 different object categories for training and evaluation.
- Foggy Cityscapes [32] is a synthetic foggy dataset which simulates fog on real scenes which automatically inherit the semantic annotations of their real, clear counterparts from Cityscapes. In particular, the experiment uses β = 0.02, which corresponds approximately to the meteorological optical range of 150 m, to remain in line with previous work.
- Sim10k [44] is a synthetic dataset consisting of 10,000 images produced from the game Grand Theft Auto V, and is excellent for evaluating synthetic to real adaptation.

Based on these datasets, these experiments evaluate CA-DINO under two widely used adaptation scenarios: (1) Normal weather to Foggy weather (Cityscapes→ Foggy Cityscapes), where the models are trained on Cityscapes and validated on Foggy Cityscapes, which aims to test domain adaptation across different weather conditions; and (2) synthetic scene to real scene (Sim10k→ Cityscapes), where Sim10k is used as source domain and Cityscapes is used as the target domain, which evaluates the shared category “Car”. Following previous works, the paper reports the results of mean average precision (mAP) with a threshold of 0.5.

### 4.2. Implementation Details

By default, ResNet-50 [30] (pre-trained on ImageNet [45]) was adopted as the backbone in all experiments. For hyper-parameters, as in DINO-4scale [18], CA-DINO uses a six-layer transformer encoder and decoder with 256 as the dimension of the hidden feature. The initial learning rate (lr) is ${1\times 10}^{-4}$ and drops lr at the 40-th epoch by multiplying 0.1, and we used the AdamW [46,47] optimizer with weight decay of ${1\times 10}^{-4}$. The weight factor $\lambda_{adv}$ and $\lambda_{coral}$ were set as 1.0.

The model was trained on NVIDIA GeForce RTX 3090 GPUs with batch size 2 (1 image each GPU × 2 GPUs) end-to-end. The software configuration adopted the deep-learning framework PyTorch 1.9, CUDA version 11.1, and Python 3.8.13. Taking Cityscapes→ Foggy Cityscapes as an example, it took about 14 h to train the model with 50 epochs.

### 4.3. Comparisons with State-of-the-Art Methods

#### 4.3.1. Normal to Foggy

**Figure 5:**
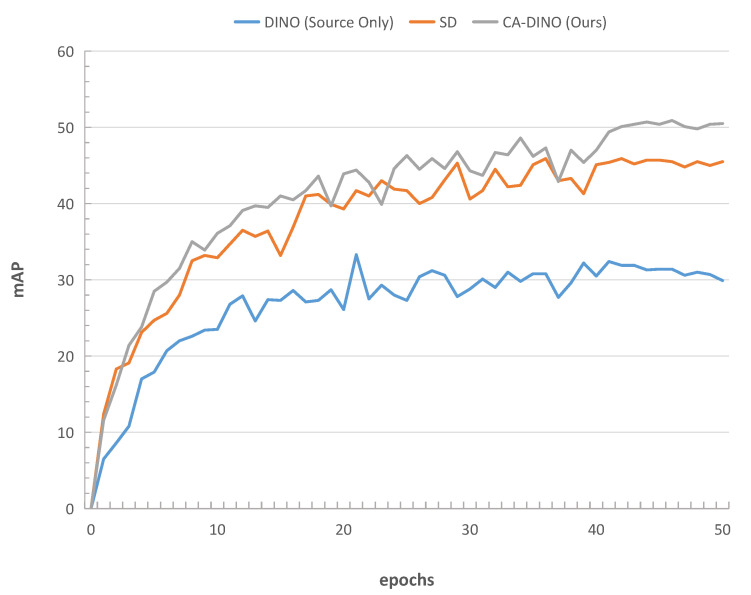
mAP curves diagram for training, Cityscapes [31] as source domain and Foggy Cityscapes [32] as target domain.

In this experiment, the Cityscapes dataset (source domain) [31] was used to train the model, which was then applied to Foggy Cityscapes (target domain) [32] for verifying the effectiveness of CA-DINO in weather scenarios. The mAP curves of the algorithm in this paper were compared with DINO [18] and the single discriminator version, as shown in Figure 5. During the training process, the performance of DINO suffers a significant decline, and the improvement in the model with the addition of epochs is negligible. When a single discriminator is introduced to be applied on the backbone for adversarial training, the performance of the model improves significantly. However, there is still a substantial gap between the model training on labeled data in the target domain. Meanwhile, CA-DINO significantly improves the cross-domain performance of DINO by 20.6 mAP, demonstrating the proposed approach’s effectiveness.

The comparisons of results with other methods are reported in Table 1. The results show that our approach is superior to traditional CNN-based domain-adaptive object detectors for most categories. In addition, the CA-DINO also performs +3.7 mAP higher than existing state-of-the-art detection transformers due to the performance of the DINO [18].

#### 4.3.2. Synthetic to Real

We used the SIM10k as the source domain and the Cityscapes as the target domain to adapt synthetic scenes to the real world. The only common category between SIM10K and Cityscapes is the car. Table 2 demonstrates that our strategy can mitigate domain shifts in various scenarios. Compared with SFA [40], the accuracy of mAP achieved a + 2.1 improvement.

### 4.4. Ablation Study

In this section, we conduct exhaustive ablation experiments on Cityscapes→ Foggy Cityscapes to determine the effect of different components in our method by adding components to DINO and comparing components before improvements as shown in Table 3.

First, by adding WROT, the mAP achieved a + 4.1% improvement. Then the simple single discriminator was added without involving an attention mechanism on the penultimate layer of the backbone; it outperforms the last one, significantly, which indicates that discriminator does help align the distributions. Further, we introduce the channel attention module to this discriminator, and the mAP is +1.3% higher than this module without attention. In addition, we separately introduce the spatial attention module on the discriminator again, which raised the mAP to 46.4. As demonstrated by the preceding results, by introducing an attention mechanism to enhance the performance of the discriminator, the discriminator is less susceptible to being deceived and the detector can learn domain-invariant features better during the adversarial learning process. Afterwards, introducing CBAM which contains a spatial-attention module and channel-attention module to the single discriminator, the mAP is +3.1% higher than the discriminator without attention and mAP reaches 48.6. By adding another discriminator with attention-enhanced for united alignment, we reach our proposed method, which yields the best performance. At the same time, we also implemented the AEDD-only version, which is slightly worse than the final model.

### 4.5. Visualization and Discussion

**Figure 6:**
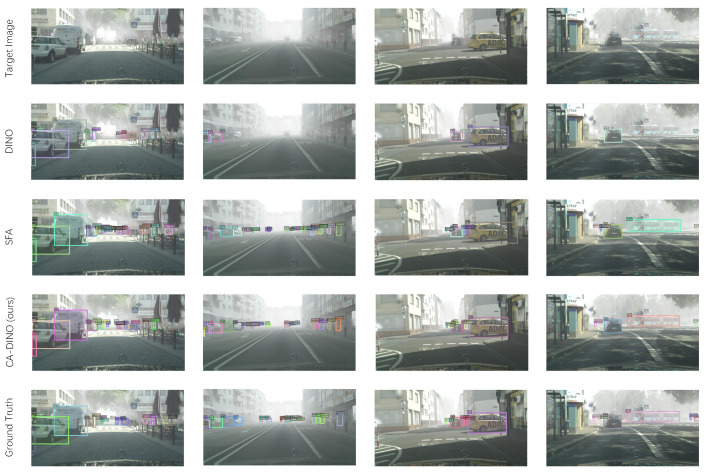
Qualitative illustration of domain-adaptive detection for Cityscapes→ Foggy Cityscapes: our method can adapt well from normal to foggy weather conditions.

To verify that our proposed model is effective, we visualized some detection results by DINO [18], SFA [40] and CA-DINO, accompanied by the ground truth. The qualitative comparison is illustrated in Figure 6. As can be seen, CA-DINO greatly minimizes false negatives, i.e., detecting objects that are not detected by other methods, proving that our proposed alignment modules may effectively decrease the domain gap and produces excellent cross-domain performance.

**Figure 7:**
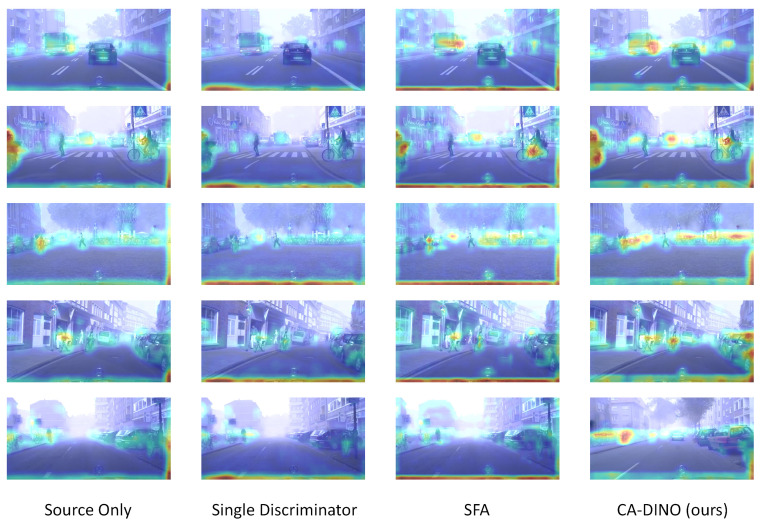
Illustration of the class activation mapping for test samples from Foggy Cityscapes.

To analyze why cascading alignment improves the detection performance, we visualize the class activation mapping [48] of backbone features extracted by the plain source model, single discriminator version, SFA and our method in Figure 7. Thanks to the well-aligned backbone, CA-DINO further facilitates attention to objects and decreases the attention on the background, especially for dense and small objects. Our model surpasses existing methods and shows advanced performance.

The outstanding performance is primarily attributed to our designed AEDD, which captures more context features at the image level. Therefore, t-SNE [29] is utilized to visualize the feature-distribution alignment of the last convolution layer of the backbone and transformer encoder and decoder from DINO and CA-DINO. Meanwhile, we visualize the single discriminator version as a comparison, as shown in Figure 2. It demonstrates that our alignment method minimizes both datasets’ domain shift. Compared to the previous two, the features extracted from the backbone, transformer, encoder and decoder by CA-DINO are well-aligned, allowing the model trained on the source domain to be effectively applied to the target domain while maintaining reasonably excellent performance.

Additionally, we attempted to implement three attention-enhanced discriminators on the backbone, and the experiments revealed that not only did we not obtain more excellent performance, but the training time was also extended. Then, we experimented with the optimal placement of these two discriminators and discovered that this has a lower influence on performance than hyperparameter adjustment. Thus, we chose the present strategy with fewer parameters. For the study, we chose CA-DINO based DINO-4scale. The parameters have 52.4 M, which includes 47 M for DINO and 5.4 M for AEDD. WROT does not contain parameters. It is noteworthy that the methods we proposed are only involved in the training stage and do not take part in inference, which allows us to infer the images at the same theoretical speed as the standard DINO, which runs at 24 FPS, similar to Faster R-CNN-FPN with the same backbone.

Segmentation [49,50] has always been a task which attracted a lot of attention in the CV community. Some recent work utilizing transformer for domain-adaptive semantic segmentation [51] have yielded positive results, while they may be specifically designed for a segmentation task. It is worthwhile to investigate how to train a segmentation model by using the trained domain-adaptive object-detection framework. One possible strategy is parameter sharing. As one of the DETR-like models, DINO can also be extended for segmentation by adding a mask head on top of the decoder outputs, just like DETR. The process is divided into two steps: first, DINO, which can be applied to the target domain, is trained by our proposed cascade-alignment framework, then all the weights are frozen and only the mask head trained on the source domain, and finally, DINO with the mask head is added and is able to infer the images from the target domain.

**Table 1:** Results on weather-adaption scenario, i.e., Cityscapes→ Foggy Cityscapes. mcycle is the abbreviation of motorcycle.

Method	Date	Detector	Person	Rider	Car	Truck	Bus	Train	Mcycle	Bicycle	mAP
DAF [24]	2018	Faster RCNN	29.2	40.4	43.4	19.7	38.3	28.5	23.7	32.7	32.0
SWDA [23]	2019	Faster RCNN	31.8	44.3	48.9	21.0	43.8	28.0	28.9	35.8	35.3
SCDA [22]	2019	Faster RCNN	33.8	42.1	52.1	26.8	42.5	26.5	29.2	34.5	35.9
MTOR [21]	2019	Faster RCNN	30.6	41.4	44.0	21.9	38.6	40.6	28.3	35.6	35.1
MCAR [52]	2020	Faster RCNN	32.0	42.1	43.9	31.3	44.1	43.4	37.4	36.6	38.8
GPA [53]	2020	Faster RCNN	32.9	46.7	54.1	24.7	45.7	41.1	32.4	38.7	39.5
UMT [54]	2021	Faster RCNN	33.0	46.7	48.6	34.1	56.5	46.8	30.4	37.3	41.7
D-adapt [39]	2022	Faster RCNN	40.8	47.1	57.5	33.5	46.9	41.4	33.6	43.0	43.0
SA-YOLO [25]	2022	YOLOv5	36.2	41.8	50.2	29.9	45.6	29.5	30.4	35.2	37.4
EPM [27]	2020	FCOS	44.2	46.6	58.5	24.8	45.2	29.1	28.6	34.6	39.0
KTNet [55]	2021	FCOS	46.4	43.2	60.6	25.8	41.2	40.4	30.7	38.8	40.9
SFA [40]	2021	Deformable DETR	46.5	48.6	62.6	25.1	46.2	29.4	28.3	44.0	41.3
OAA + OTA [56]	2022	Deformable DETR	48.7	51.5	63.6	31.1	47.6	47.8	38.0	45.9	46.8
CA-DINO (Ours)	2022	DINO	**54.5**	**55.6**	**69.1**	**36.2**	**57.8**	42.8	**38.3**	**50.1**	**50.5**

**Table 2:** Results on synthetic to real adaptation scenario, i.e., Sim10k→ Cityscapes. mcycle is the abbreviation of motorcycle.

Method	Date	Detector	Car AP
DAF [24]	2018	Faster RCNN	41.9
SWDA [23]	2019	Faster RCNN	44.6
SCDA [22]	2019	Faster RCNN	45.1
MTOR [21]	2019	Faster RCNN	46.6
CR-DA [57]	2020	Faster RCNN	43.1
CR-SW [57]	2020	Faster RCNN	46.2
GPA [53]	2020	Faster RCNN	47.6
D-adapt [39]	2022	Faster RCNN	49.3
SA-YOLO [25]	2022	YOLOv5	42.6
EPM [27]	2020	FCOS	47.3
KTNet [55]	2021	FCOS	50.7
SFA [40]	2021	Deformable DETR	52.6
CA-DINO(Ours)	2022	DINO	**54.7**

**Table 3:** Results on ablation study. mcycle is the abbreviation of motorcycle. SD is a single discriminator, cam-SD and sam-SD represent SD with channel attention module, and spatial attention module has been introduced, respectively. AESD is attention-enhanced single discriminator. Oracle is the result of DINO training with labeled target domain dataset.

Method	Person	Rider	Car	Truck	Bus	Train	Mcycle	Bicycle	mAP
DINO [18]	38.2	38.2	45.2	18.2	31.9	6.0	22.3	37.9	29.9
+WROT	43.0	46.6	58.4	18.7	32.2	11.3	23.3	38.3	34.0
+SD +WROT	51.1	52.6	64.0	26.4	51.1	36.0	35.5	47.4	45.5
+cam-SD +WROT	51.8	55.0	64.5	32.6	51.7	37.8	31.8	49.0	46.8
+sam-SD +WROT	52.0	52.9	63.8	27.1	51.2	43.9	32.5	48.0	46.4
+AESD +WROT	51.7	54.7	67.5	29.7	52.0	44.0	40.3	49.1	48.6
+AEDD	55.0	55.0	68.6	32.1	58.5	34.2	37.9	50.8	49.0
+AEDD +WROT	54.5	55.6	69.1	36.2	57.8	42.8	38.3	50.1	**50.5**
oracle	58.4	54.8	77.2	36.9	56.5	39.4	40.8	51.2	51.9

## 5. Conclusions

In this paper, we were devoted to enhancing the cross-domain performance of DINO for unsupervised domain adaptation. Specifically, CA-DINO includes attention-enhanced double discriminators (AEDD), which are proposed to extract more domain-invariant features and weak-restraints on category-level token (WROT) for minimizing the difference in second-order statistics between the source and target domain. Numerous experiments and ablation studies have also demonstrated the effectiveness of our method. Although CA-DINO has excellent performance, one GPU can only carry one batch in the experiments. Our method requires more memory than previous work and takes longer to train. The introduction of WROT largely alleviates the instability brought by adversarial training. However, the model’s training is still accompanied by a slight perturbations in some scenarios, which makes the adjustment of hyperparameters particularly difficult. Balancing performance and stability is the next important direction for us to explore.

## Data Availability

Publicly available datasets were analyzed in this study. This data can be found here: https://www.cityscapes-dataset.com/accessionnumber (Cityscapes, Foggy Cityscapes) and https://fcav.engin.umich.edu/projects/driving-in-the-matrix (Sim10k). Both accessed on 1 May 2022.
